# Pt and RhPt dendritic nanowires and their potential application as anodic catalysts for fuel cells†

**DOI:** 10.1039/c9ra04801d

**Published:** 2019-10-02

**Authors:** Daniel K. Kehoe, Sarah A. McCarthy, Luis Romeral, Michael G. Lyons, Yurii K. Gun'ko

**Affiliations:** School of Chemistry, Amber & CRANN Institute, Trinity College Dublin Dublin 2 Ireland igounko@tcd.ie; BEACON, Bioeconomy Research Centre, University College Dublin Dublin 4 Ireland

## Abstract

Fuel cells have a number of benefits over conventional combustion-based technologies and can be used in a range of important applications, including transportation, as well as stationary, portable and emergency backup power systems. One of the major challenges in this field, however lies in controlling catalyst design which is critical for developing efficient and cost-effective fuel cell technology. Herein, for the first time, we report a facile controlled synthesis of Pt and RhPt dendritic nanowires using ultrathin AuAg nanowires as sacrificial templates. These dendritic nanowires exhibit remarkable catalytic performance in the elecrochemical oxidation of methanol and formic acid. In particular, the RhPt dendritic nanostructures show very high resistance to catalyst poisoning in methanol oxidation. This research demonstrates the advantages of using bimetallic dendritic nanostructures and we believe that these materials and electrocatalytic studies are important for further advancement of fuel cell research and technology.

## Abstract 2

Fuel cells have a number of benefits over conventional combustion-based technologies and can be used in a range of important applications, including transportation, as well as stationary, portable and emergency backup power systems.

## Graphical abstract

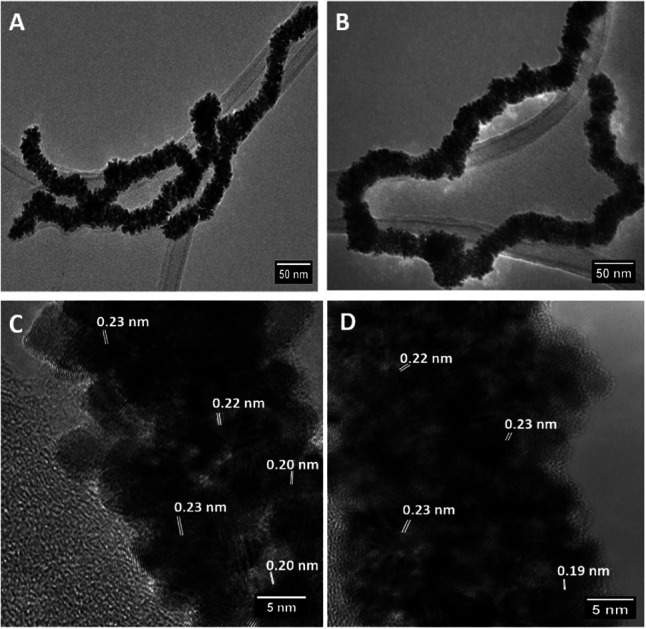

## Introduction

Fuel cells have long been recognised as very promising candidates for providing clean and sustainable energy for the future. Methanol in particular has become a notable fuel of choice as it is relatively easy to transport and offers appreciable current densities.^1^ However, among the biggest obstacles for the commercialization of fuel cells is the cost of the catalyst, which is commonly Pt based, and inefficiencies arising from catalytic poisoning.^2^ The poisoning issue is particularly significant for methanol based fuels cells as in addition to CO adsorption, they also have a tendency to undergo crossover poisoning, in which methanol crosses over the polyelectrolyte membrane and poisons the cathode.^3–5^ In an attempt to mitigate this issue researchers have looked for various alternative types of fuels. To this end, formic acid stands as a promising substitute to methanol as in addition to low toxicity, it is also less susceptible to undergo fuel crossover poisoning and has shown fast oxidation kinetics.^6,7^ It has been established that the electro-oxidation of formic acid occurs *via* a parallel dual pathway mechanism: the direct path, involving the dehydrogenation of adsorbed formate producing CO_2_ resulting in no poisoning, and the indirect path whereby CO poisons are generated following the oxidation of adsorbed intermediates.^8–11^ Recently, it has emerged that the indirect pathway is only a minor path and does not contribute significantly to the overall current.

While the exact mechanism of these pathways remains unclear, Cuesta *et al.*^12^ have shown that absorbed formate plays a key role in both cases. The choice of catalyst has the most significant influence in the electro-oxidation of the fuel, thus catalyst design such as morphology and composition are of great importance.^13–16^ Researchers have particularly focused on alloying of metal catalysts as an amenable approach for not only reducing the cost of catalysts but also enhancing catalytic performance.^17–20^ In addition RhPt nanostructures,^21^ RhPt ultrathin nanowires (NWs)^22^ and other alloys such as PtPd dendrites^23^ and PtAg ultrathin NWs^24^ have also showed remarkable catalytic performances by virtue of their morphologies and compositions.

Over the last few years there has been a considerable drive to develop methods to synthetically control the morphology of nanomaterials. It is well accepted that the size and morphology of nanomaterials can have a significant effect on their catalytic and even optical properties.^25–27^ One of the most effective methods for achieving control in nanomaterial design is *via* template based synthesis. Typical examples include the use of soft templates such as DNA^28,29^ and hard templates such as aluminium oxide.^30–32^ A major disadvantage of this approach however, is the post synthesis template removal. Recently sacrificial templating has been realized as route to overcoming this problem.^33,34^ This method relies on the galvanic replacement of the template, for the purpose of our work specifically NWs, resulting in dissociation of the template and its one dimensional (1D) morphology being preserved in the product.^35,36^ Work by Liang *et al.*^37^ demonstrated the use of ultrathin Te NWs as sacrificial templates for the synthesis of ultrathin Pt nanotubes (NTs) and Pd NWs. The authors showed that the oxidation state of the metal precursor has a significant impact on the product with Pt^2+^ resulting in NTs and with Pt^4+^ in NWs. Ultrathin Te NWs have since been used as templates in the synthesis of a variety of novel 1D nanomaterials such as; ultrathin PtCu NWs,^38^ AuPt NTs,^35^ PtAgTe NWs^39^ and PtPdRuTe NTs^40^ with each of these nanomaterials exhibiting interesting catalytic performances particularly for fuel cell applications.

Ag NWs have also been used as sacrificial templates in the synthesis of Pt based nanomaterials, most notably producing hollow NT structures.^41,42^ Chen *et al.*^43^ demonstrated the synthesis of double walled AuPt hollow NTs through a 2 step process. Firstly, Au undergoes a galvanic reaction with the Ag NWs to afford hollow Au NTs with a thin sheath of Ag on the surface, these subsequently undergo a further galvanic reaction with Pt producing AuPt hollow NTs. Interestingly the inner wall of these NTs was made of Au while the outer wall was made up of Pt. This multi-step approach highlights the versatility of templating as a controlled way of producing complex nanostructures.

In addition to Ag NWs, ultrathin Au NWs have recently emerged as promising sacrificial templates. Hong *et al.*^44^ showed that these NWs can be used to produce dendritic NWs composed of AuPt and AuPtCu with considerable catalytic activity for methanol oxidation. While the cost of the template material is still of concern particularly for large scale applications, researchers have employed Cu NWs as a cheaper alternative for sacrificial templating. This has seen the development of various alloys such as AuCu,^45,46^ PtCu^45,47^ and AuPtCu NTs.^48^ Here, we present for the first time the use of AuAg ultrathin NWs as sacrificial templates in the synthesis of Pt and RhPt dendritic NWs and their use as anodic catalysts for methanol and formic acid oxidation.

## Experimental

### Characterisation techniques

Transmission electron microscopy (TEM), high resolution transmission electron microscopy (HR-TEM), high angle annular dark field-scanning transmission electron microscopic (HAADF-STEM) and energy dispersive X-ray spectroscopy (EDX) were performed using a FEI Titan. UV-Vis spectra were recorded on a LAMBDA 1050 UV/vis/NIR spectrometer using a quartz cuvette with a 1 cm pathlength. X-ray diffraction (XRD) analysis was performed on Bruker D2 Phaser diffractometer. Cyclic voltammetry and *I*–*t* curves were recorded using a CH instruments electrochemical workstation. Electrochemical impedance spectroscopy (EIS) was carried out on a Zahner Elektrik IM-6.

### Synthesis of ultrathin AuAg nanowires

Ultrathin AuAg NWs were synthesised using our previously reported procedure.^49^ 1 mL, 500 mM of polyvinylpyrrolidone (PVP), HAuCl_3_·*n*3H_2_O (200 μL, 50 mM) and AgNO_3_ (200 μL, 50 mM) were dissolved in dimethylformamide (DMF) (8 mL). The solution was vortexed for 2 minutes and an aqueous solution of ascorbic acid (1 mL, 400 mM) was then injected into the solution. The mixture was vortexed for a further 30 s and then left standing for 18 h at room temperature. After aging, the solution was then diluted by a factor of 20 with water. The resulting grey solution was then centrifuged twice (9000 rpm, 35 min) and the precipitate was re-dispersed in water.

### Templated synthesis of Pt and RhPt dendritic nanowires

PVP (30.3 mg, 55 000 wt) was dissolved in water (14 mL). Ultrathin AuAg NWs (1 mL, 0.35 mg mL^−1^) were then added and the solution was heated to 80 °C. RhCl_3_ (710.5 μL, 19 mM) and H_2_PtCl_4_ (2.7 mL, 5 mM) were added sequentially and the solution was stirred for 1 min. In the case of the Pt dendritic NWs only 2 equivalents of H_2_PtCl_4_ were added as the metal precursor. An aqueous solution of ascorbic acid (1 mL, 1 mM) was then added and the solution was left heated at 80 °C for 1 hour. The solution was then cooled to room temperature and acetone was added. The final product was then purified by centrifugation (9000 rpm, 20 min) and re-dispersed in water.

### Pt and RhPt dendritic nanowires for electrocatalytic oxidation of methanol and formic acid

All electrochemical analysis was performed using a conventional three electrode electrochemical cell at 25 °C. A glassy carbon electrode was used as the working electrode, Ag/AgCl as the reference electrode and Pt wire as the counter electrode, respectively. For all analysis the working electrode was modified with the Pt or RhPt NWs by drop casting from stock solutions and allowing to dry in air. Typically 10 μg of catalyst in each case was used. Nafion (5 μL) 0.1 wt% was then drop cast onto the modified electrode and allowed to air dry. In the case of methanol, perchloric acid was used as the electrolyte. While for formic acid oxidation, H_2_SO_4_ was used as the electrolyte. The cyclic voltammograms (CVs) were obtained in nitrogen-saturated solutions and the potential was measured from −0.25 to 1.05 V (Ag/AgCl) at a scan rate 50 mV s^−1^ in all cases. CV measurements for the oxidation of each substance were carried out in a solution of 1 M methanol or 0.5 M formic acid with a 0.5 M of electrolyte.

Electrochemical impedance spectroscopy (EIS) measurements were performed using a three-electrode electrochemical cell with wires of 10 cm length. The electrochemical impedance response were recorded over a range of potentials associated with active oxygen evolution, typically in the range 0.42–0.58 V (*vs.* Ag/AgCl) with an AC signal amplitude of 5 mV. The frequency span was from 1 MHz down to 50 mHz. All the EIS measurements were temperature controlled using a F12-ED Refrigerated/Heating Circulator and kept constant at 25 °C.

## Results and discussion

**Fig. 1:**
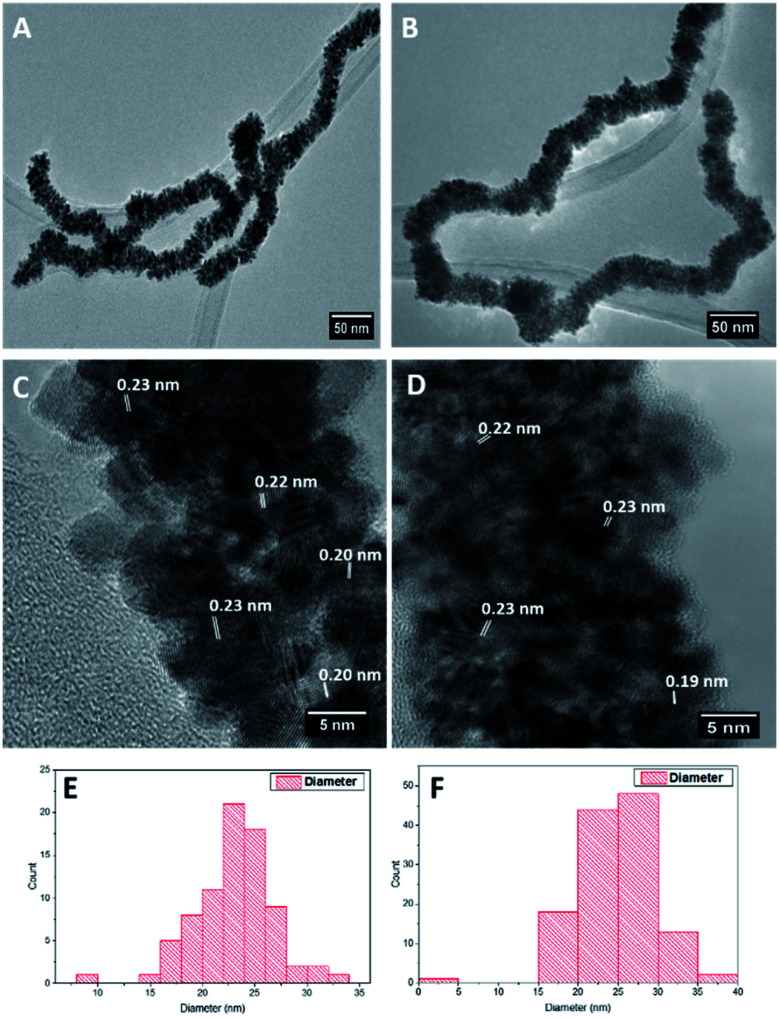
TEM images (A and B), HR-TEM images (C and D), size distribution analysis (E and F) of Pt (left column) and RhPt (right column) dendritic nanowires.

Pt and RhPt dendritic NWs were synthesised using ultrathin AuAg NWs as sacrificial templates (refer to Fig. S1† for characterisation of template NWs). Briefly PVP and the ultrathin AuAg NWs were mixed together in water and heated to 80 °C. In the case of the RhPt dendritic NWs, RhCl_3_ and H_2_PtCl_4_ were added sequentially (1 : 1 molar ratio), while for Pt dendritic NWs only H_2_PtCl_4_ (2 eq.) was added. Ascorbic acid was then added after 1 min and the mixture was left under heating at 80 °C for 1 hour. HR-TEM analysis (Fig. 1A and B) shows that the ultrathin AuAg NWs performed as effective templates producing high yields of product in both cases. The resulting structures consist of an assembly of anisotropic nanoparticles with a NW formation. These structures closely resemble the AuPt and AuPtCu dendritic NWs produced by Hong *et al.*^44^ using ultrathin Au NWs as templates. Size distribution analysis (Fig. 1E and F) shows that both products are of similar size ranging up to microns in length and with average diameters of 23.7 and 24.7 nm for the Pt and RhPt dendritic NWs, respectively. The Pt dendritic NWs have *d*-spacing values of 0.23 and 0.20 which correlates with the 111 and 200 planes of face-centered cubic (FCC) Pt respectively. In the case of the RhPt dendritic NWs, HR-TEM (Fig. 1C and D) revealed similar *d*-spacing values of 0.19 and 0.23 nm which correlates with the 111 and 200 planes of FCC Rh and Pt. The size of the nanoparticles in these dendritic structures was found to range from 2 to 8 nm for both the Pt and RhPt dendritic NWs. UV-vis analysis (Fig. S2 in ESI†) further shows broad absorbance profiles in both cases with Pt in particular exhibiting a peak 290 nm which is typical for these nanoparticles.^50^

**Fig. 2:**
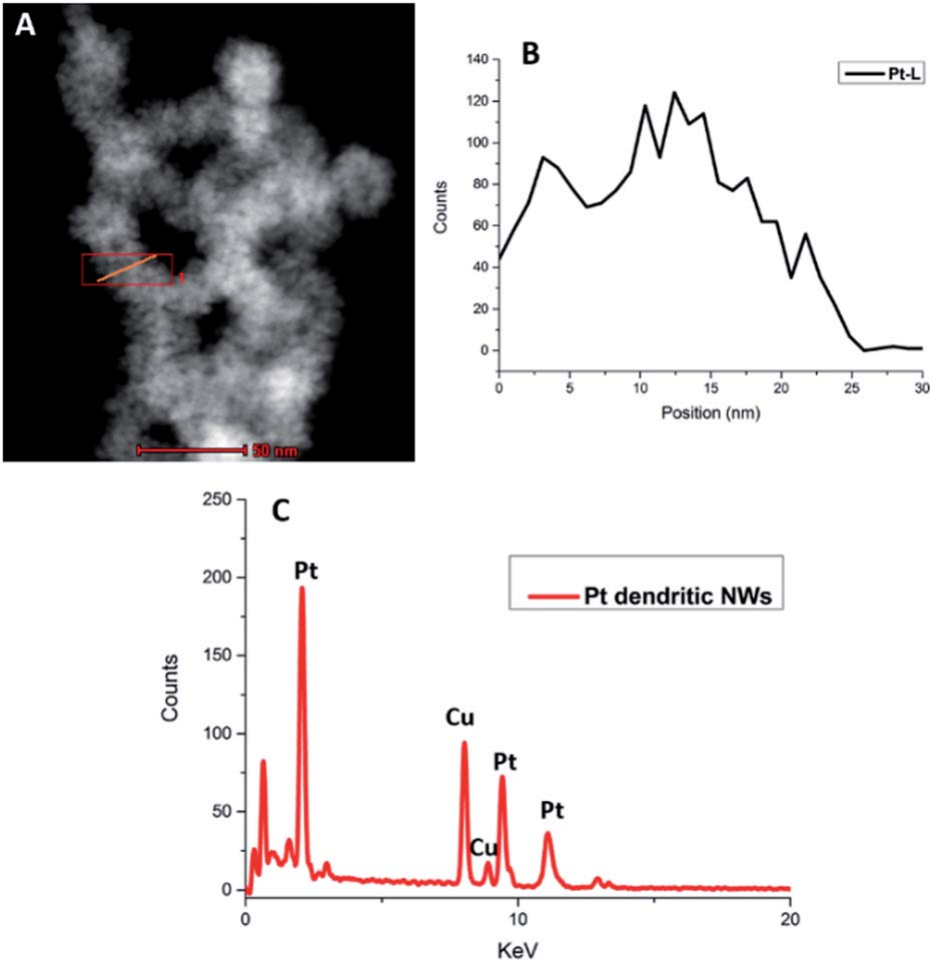
HAADF STEM image highlighting region of interest (A), EDX map of Pt L line (B) and corresponding EDX spectrum (C) of Pt dendritic NWs.

HAADF-STEM EDX analysis of the Pt dendritic NWs (Fig. 2) confirms that the structure is composed entirely of Pt. The EDX spectrum shows the characteristic L and M peaks of Pt at 9.44 and 2.04 keV, respectively. The additional peaks present are due to the Cu from the TEM grids used for this analysis. In addition elemental analysis of the RhPt dendritic NWs also shows that they are alloy of Rh and Pt with a Pt : Rh ratio of 90 : 10. The EDX spectrum shows the characteristic L and M peaks of Pt at 9.44 and 2.04 keV respectively and the K peak of Rh at 20 keV. The additional peaks present are again due to the Cu from the TEM grids used for this analysis (ESI, Fig. S3†). Most importantly in both cases our elemental analysis did not show the presence of any Au or Ag from the template in either nanostructure. This confirms that the NWs are performing as sacrificial templates and are completely removed during the synthesis. This is a striking result as unlike in the case of Hong *et al.*,^44^ where Au from the Au NW template remains in the final product in our case it does not. The reason for this may be due to the presence of Ag in our AuAg NW alloy facilitating the complete dissociation of the template following the galvanic reaction with the metal precursors.

**Fig. 3:**
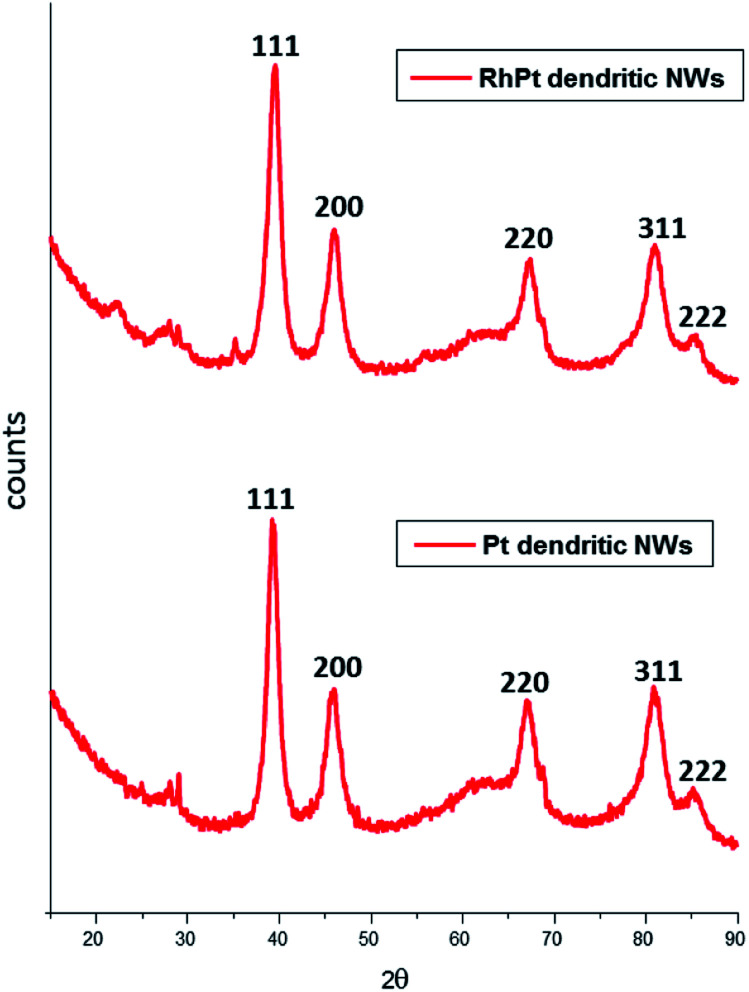
XRD analysis of Pt (bottom) and RhPt (top) of dendritic nanowires.

The XRD patterns of both dendritic NWs (Fig. 3) are very similar with Pt exhibiting diffraction peaks corresponding to the (111), (200), (220) and (311) planes respectively for FCC Pt (JCPDS-01-117). This observation is common for noble metal nanomaterials of this kind. Furthermore, the peak intensity ratio of the (111) compared to (200) and (220) for the Pt dendritic NWs, was determined to be 1.62 and 1.92 respectively. This analysis indicates that the dendritic NWs preferentially grow along the (111) plane. This is to be expected as we demonstrated in our previous work,^49^ that the (111) facet of our AuAg NWs is the most dominant.

**Fig. 4:**
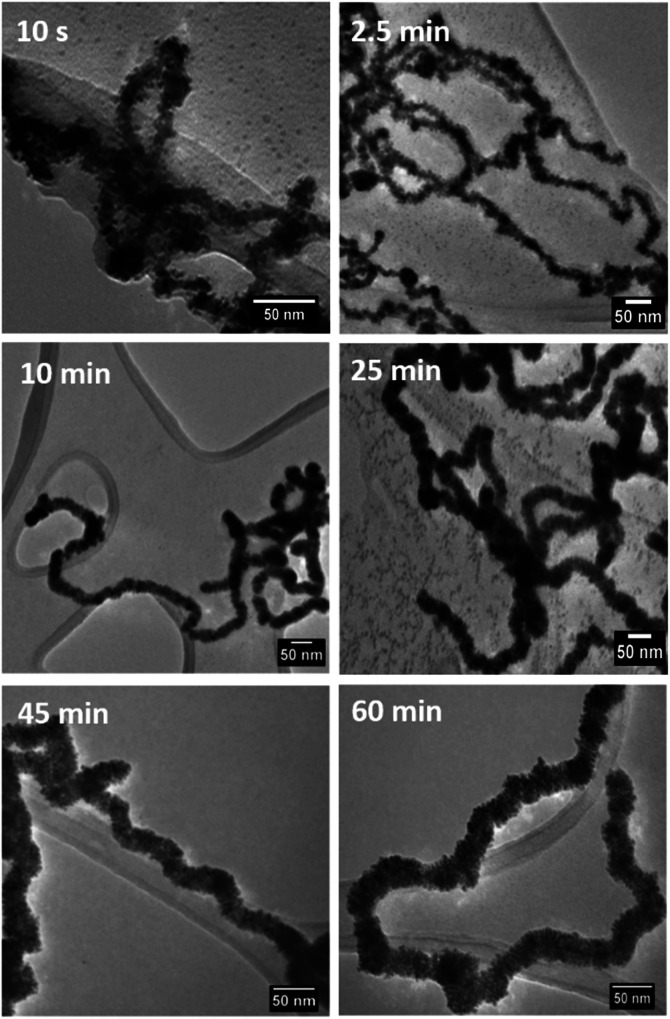
TEM analysis monitoring the formation of RhPt dendritic nanowires over time.

We investigated the formation of the dendritic NWs by taking aliquot from the reaction mixture at various times intervals, following the addition of ascorbic acid, and prepared them quickly on lacy carbon TEM grids for TEM analysis. This was performed only for the RhPt dendritic NWs (Fig. 4).

**Fig. 5:**
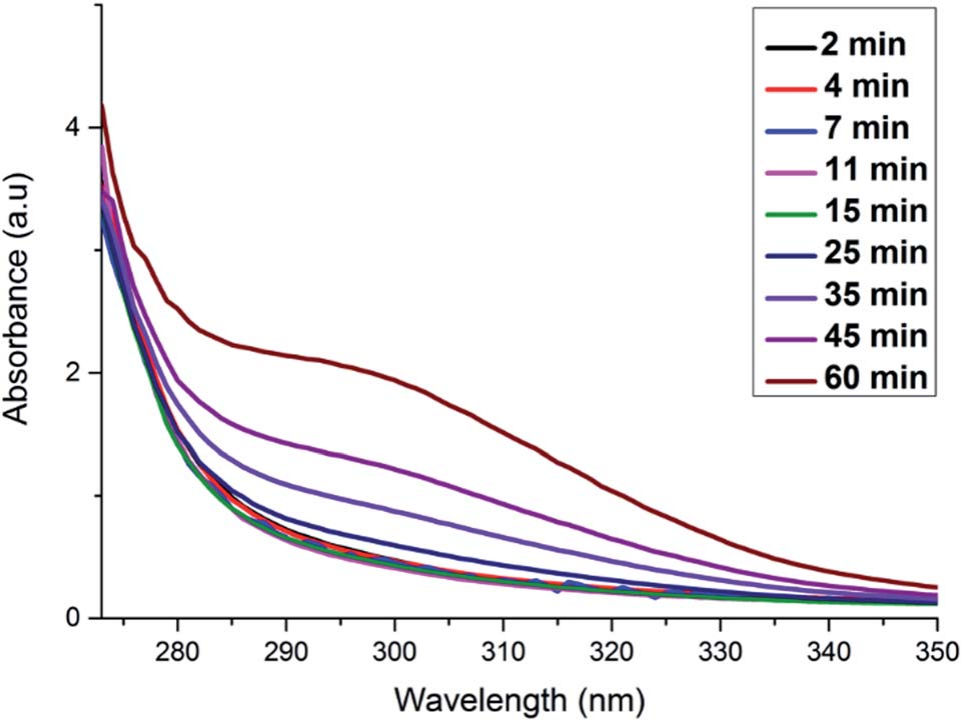
UV-vis analysis monitoring the formation of RhPt dendritic nanowires over time.

Our TEM analysis clearly shows that within the 10 s of adding ascorbic acid the dendritic NWs begin to form, as noted by the nanoparticle aggregates formed on the template. By 2.5 min we see complete formation of dendritic NWs with an average diameter of 15.1 nm (Fig. S4†). After 10 min the dendritic NWs remain of a similar size with an average diameter of 15.5 nm (Fig. S5†). The dendritic NWs gradually increase in thickness reaching a final thickness of typically 25 nm after 25 min. The diameter remains unchanged upon completion of the reaction at 60 min (Fig. S7†). In addition to TEM, we also performed UV-vis analysis to monitor the evolution of these nanostructures. As shown in Fig. 5 over the course of this reaction we see the gradual appearance of a broad absorbance band centred around 300 nm which is due to the increase of the concentration of RhPt nanoparticles in solution. This is further supported by our control studies discussed below.

**Fig. 6:**
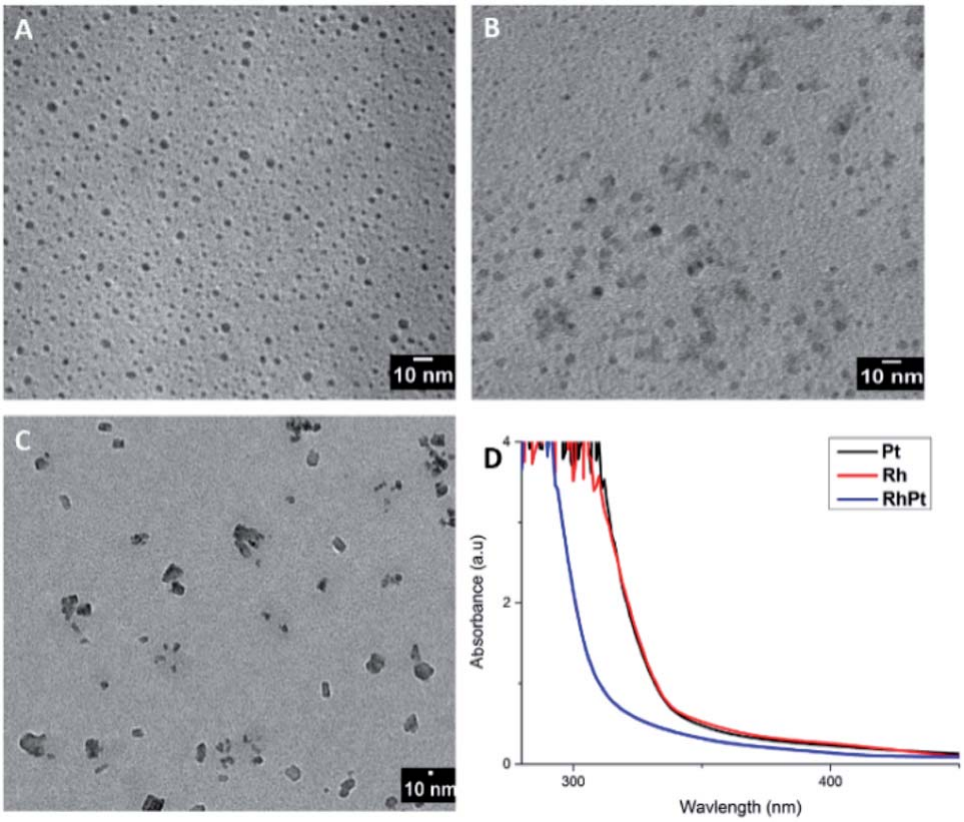
TEM images of Rh, Pt and the RhPt controls (A–C) in the absence of templates and associated UV-vis spectra (D).

We performed a control study with Rh, Pt and a mixture of Rh and Pt (1 : 1 molar ratio) precursors in the absence of the AuAg NW template. The concentration of the metal precursors was kept the same in all cases (27 μmol) and the products were analysed by TEM analysis and UV-vis spectroscopy as shown in Fig. 6. As expected, in the absence of the AuAg NW templates no dendritic NWs are formed. In all cases only nanoparticles were produced from each control. The average diameters were 2.3, 2.42 and 22.7 nm for the Pt, Rh and RhPt nanoparticles respectively. This result was similarly observed by Tu *et al.*^51^ in case of Pt and Rh and by Lee *et al.*^21^ for the Rh and Pt mixture. Regarding the UV-vis analysis, each control shows broad absorbance bands centred at 300 nm, which is common for these nanoparticles. Thus, our control study clearly confirms that the AuAg NWs are performing as templates and are really necessary to produce the dendritic NWs structure.

We also investigated the role of the capping agent, in this case PVP, in the formation of the dendritic NWs. In this study we performed the reaction in the absence of PVP and focused only on RhPt dendritic NWs. As shown in Fig. S8 (ESI†) the main product was predominantly nanoparticles. While the presence of dendritic NW like structures were noted they however lack shape control and uniformity. Thus, this study highlights that PVP is also necessary to control the shape and quality of the resulting dendritic nanostructure. In addition, this study further supports the role of the AuAg NWs as sacrificial templates.

**Fig. 7:**
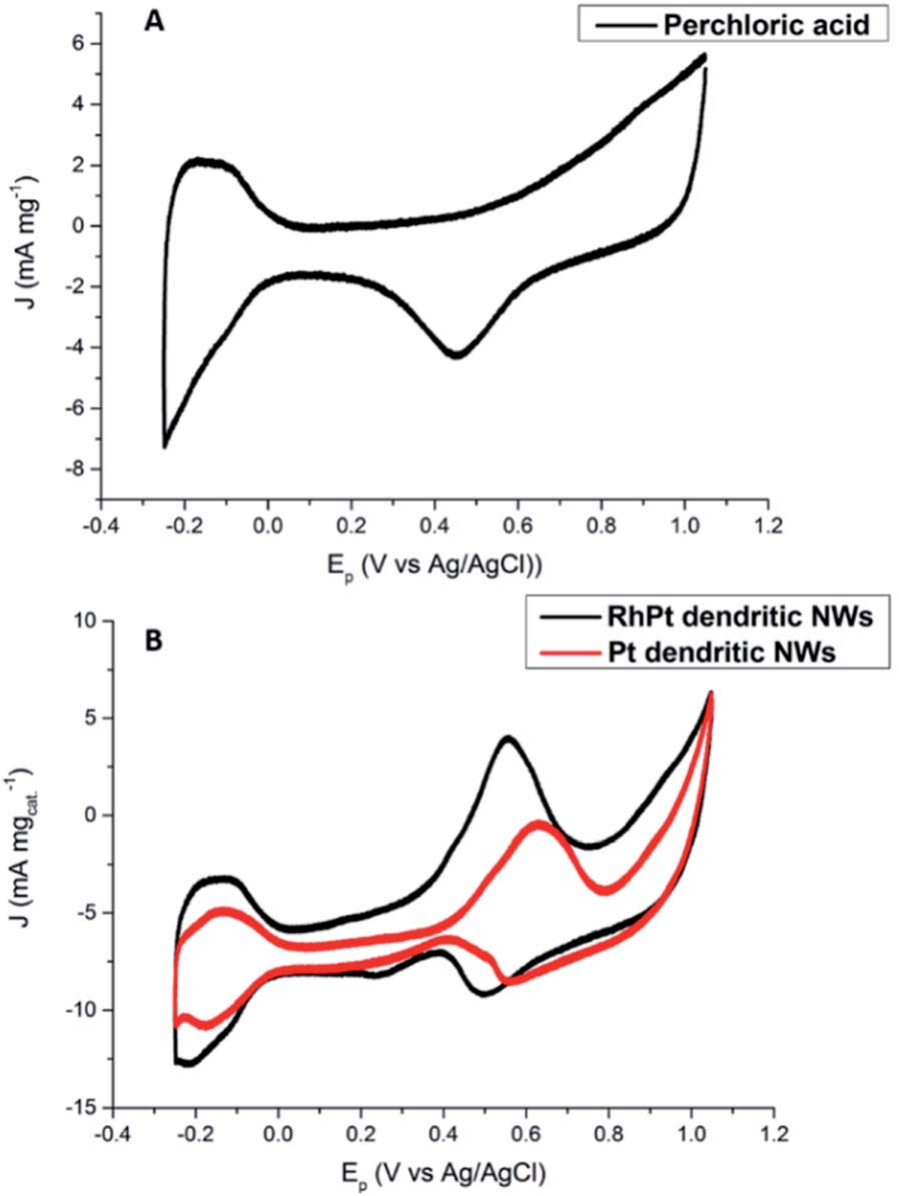
CV analysis of 0.5 M perchloric acid (A) and 1 M methanol + 0.5 M perchloric acid (B) at 50 mV s^−1^ for Pt and RhPt dendritic NWs *versus* Ag/AgCl reference electrode.

The electro-catalytic activities of the Pt and RhPt dendritic NWs were assessed by cyclic voltammetry (CV) in a 0.5 M perchloric acid solution containing 1 M methanol (Fig. 7). For each electrochemical test a 10 μg of loading of catalyst was drop cast onto the glassy carbon electrode (GCE). The RhPt dendritic NWs produce a current density 1.1 times greater than the Pt dendritic NWs. Furthermore the peak potential from the RhPt dendritic NWs is more negative (0.55 V) than the Pt dendritic NWs which have a peak potential at 0.63 V. These results highlight that alloying Rh with Pt enhances the catalytic performance, most likely due to synergistic effects between the 2 metals in the alloy. In addition both these catalysts also exhibit 2 peaks: the peak in the forward scan is due to the oxidation of the methanol while the peak in the reverse scan is due to the oxidation of surface poisons. The larger the ratio of the forward peak (*J*_f_) to the reverse peak (*J*_b_), the greater the electrodes poison-resistance ability. In our case *J*_f_/*J*_b_ for the Pt and RhPt dendritic NWs was found to be 2.3 and 2.9 respectively which are considerably high poisoning resistances. While a “bifunctional mechanism” is typically used to describe the enhanced performance for PtM alloys (M = Ru, Sn and Rh) particularly for methanol oxidation, Sheng and co-workers^52^ recently showed through density functional theory (DFT) calculation that in the case of RhPt it is more complex. The authors showed that Rh may play a more active role in the initial C–H bond breaking in the case of methanol rather than just forming OH species. Comparison with other Pt containing catalyst shows that these dendritic NWs offer considerable catalytic performance with remarkable poison resistance (Table 1). Electrochemical impedance spectroscopy (EIS) was further carried out on the RhPt dendritic nanowires in 1 M methanol and 0.5 M perchloric acid solution in order to further understand the methanol oxidation reaction using this catalyst. The Nyquist plot (see Fig. S9 in ESI†) shows that below 0.50 V only straight lines are observed indicating that no methanol oxidation occurs as expected from CV analysis.^53^ As the potential increases (0.52–0.58 V) we see that the EIS form a semi-circle in the lower frequency range, which is associated with charge transfer resistance, while at higher frequencies particularly at 0.58 V diffusion processes (Warburg impedance) begin to occur as noted by the slight sloping straight line.^54–58^ This indicates that the diffusion of reactants to the catalyst surface is the rate determining step. In addition the absence of a “pseudo-inductive” loop which is common for Pt based catalyst for this reaction implies that the binding of CO to the surface may be weak and this compliments the large poison resistance noted by this catalyst.^59–61^

**Table 1:** Electro-oxidation of methanol by various Pt based catalyst in acidic medium

Catalyst	*E*_p_ (V)	*J*_f_/*J*_b_
Au/Pt dendritic NWs^44^	−0.10 (*vs.* Ag/AgCl)	1.25
Au/PtCu dendritic NWs^44^	−0.075 (*vs.* Ag/AgCl)	1.51
RhPt nanodendrites^21^	0.62 (*vs.* Ag/AgCl)	3.02
Pd_75_Pt_25_ nanodendrites^23^	0.594 (*vs.* SCE)	*ca.* 0.86
Ultrathin Pt_3_Co NWs^62^	0.8 (*vs.* Ag/AgCl)	0.88
Ultrathin Pt_4_Pb NWs^19^	0.68 (*vs.* Ag/AgCl)	1.09
Pt_66_Ni_27_Ru_7_ nanodendrites^63^	*ca.* 0.64 (*vs.* SCE)	1.12
Ultrathin Pt_3_Cu wavy NWs^64^	0.64 (*vs.* SCE)	1.67
Pt_3_Cu triangular pyramid caps^65^	0.95 (*vs.* RHE)	1.55
Pt dendritic NWs (this work)	0.63 (*vs.* Ag/AgCl)	2.3
PtRh dendritic NWs (this work)	0.55 (*vs.* Ag/AgCl)	2.9

**Fig. 8:**
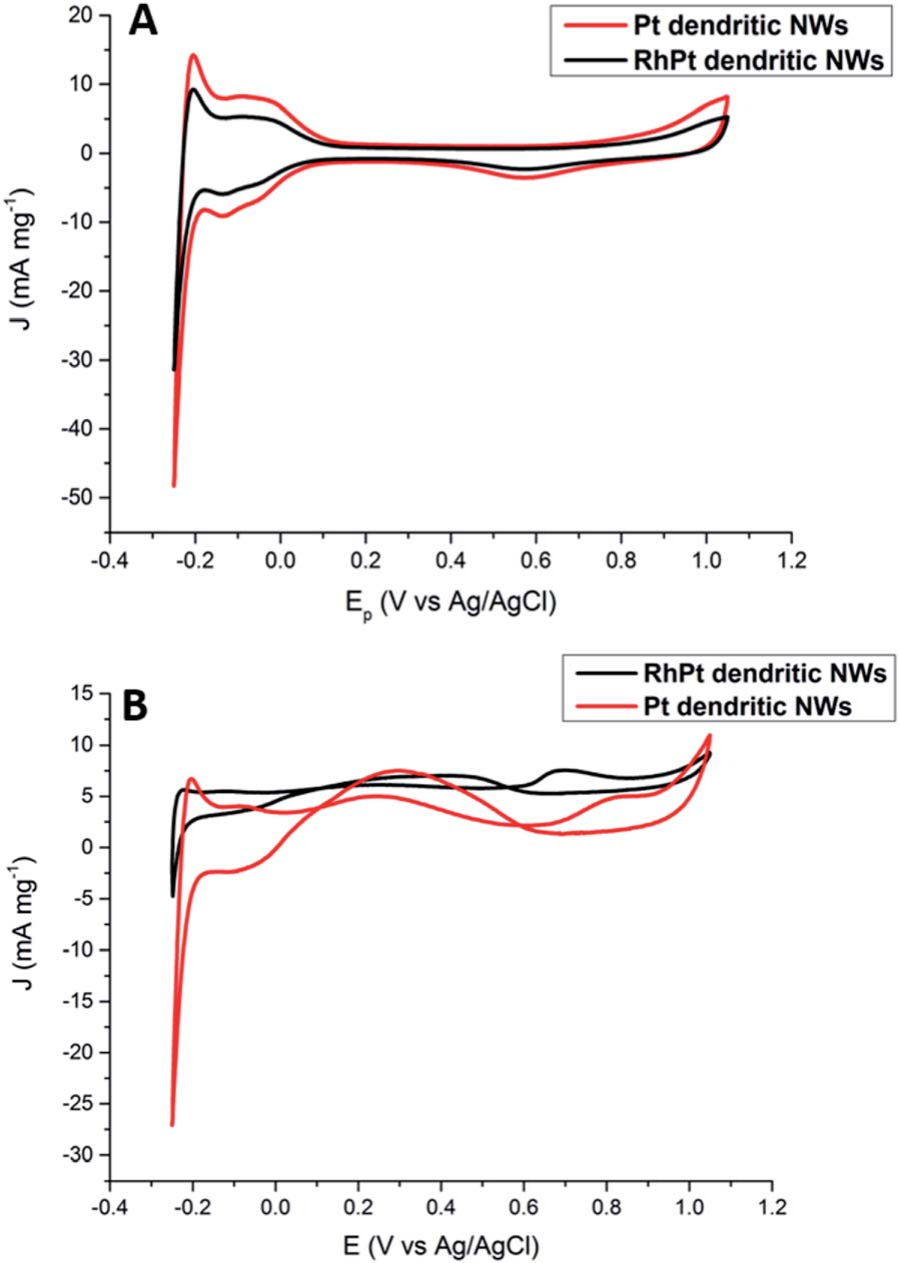
CV analysis of 1 M H_2_SO_4_ (A) and 0.5 M formic acid + 1 M H_2_SO_4_ (B) solutions at 50 mV s^−1^ for Pt and RhPt dendritic NWs *versus* Ag/AgCl reference electrode.

Regarding, formic acid oxidation, we assessed the electro-catalytic activities of our Pt and RhPt dendritic NWs by CV in a 1 M H_2_SO_4_ solution containing 0.5 M formic acid (Fig. 8). The RhPt dendritic NWs again out-perform the Pt dendritic NWs producing a 1.53 times larger current density at more a negative peak potential (*Δ* 0.14 V).

In addition, both these catalysts exhibit 2 peaks. Since the direct pathway has been recognised as the predominant pathway, it is generally accepted that the peak in the forward scan (*J*_f_) is due to the oxidation of absorbed formate while the peak in the reverse scan (*J*_b_) is due to oxidation of surface poisons. *J*_f_/*J*_b_ for the Pt and RhPt dendritic NWs was found to be 1.5 and 3.1 respectively. Comparison of both these dendritic NWs with various 1D noble metal catalysts (Table S1†) highlights that our RhPt dendritic nanostructures in particular are highly resilient to poisoning. These results also further highlight the benefit of alloying for enhancing catalytic performance. Using chrono-amperimetric *I*–*t* curves over a 4000 s period (Fig. S10†), it was further found that both dendritic NWs have good long term catalytic stability.

**Fig. 9:**
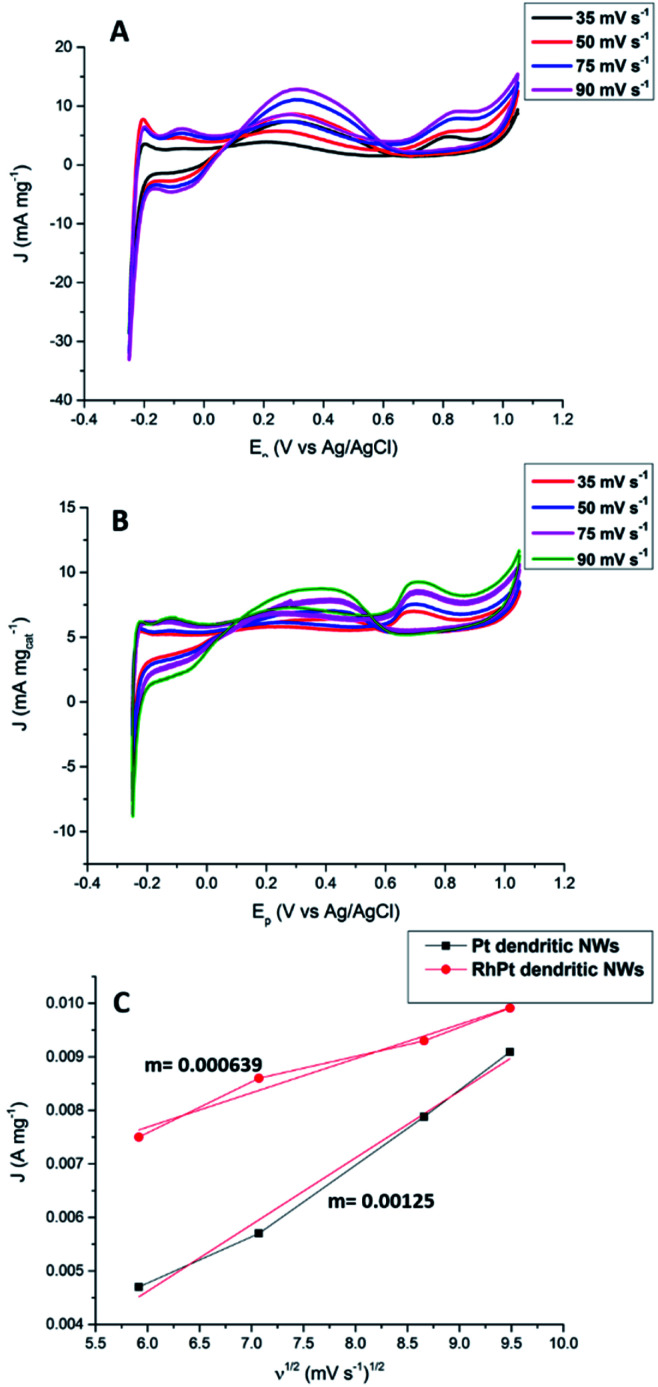
CV analysis of formic oxidation with Pt (A) and RhPt (B) dendritic NWs at various scan rates *versus* Ag/AgCl reference electrode, and corresponding plot of the forward peak current density *versus* the square root of the scan rate (C).

We also further investigated the mechanism for this electro-oxidation process by varying the scan rate in our CV analysis. According to results shown in Fig. 9 as the scan rate is increased the peak current densities also rise for both the Pt and RhPt dendritic NWs. A plot of the peak current density in the forward scan with respect to square root of the scan rate shows a linear relationship in both cases. This indicates that reaction process is diffusion rate limited.^66^ Interestingly the Pt dendritic NWs exhibited greater kinetics compared to the RhPt dendritic NWs as noted by the larger slope. As mentioned earlier Rh has been shown to play a complex role in these oxidation processes and this may account for the slower kinetics.^52^ Nevertheless, the RhPt alloy still out performed the Pt monometallic counterpart producing a higher current density at a lower potential. In the future we plan to perform a more detailed mechanistic study of this oxidation process with both of these catalysts.

## Conclusions

Thus, for the first time we employed ultrathin AuAg NWs as sacrificial templates for the synthesis of new dendritic 1D nanomaterials. Using TEM analysis it was found that initial galvanic reaction for the synthesis of the RhPt dendritic NWs occurs rapidly, followed by the gradual increase in the thickness in the dendritic NWs. In addition we further demonstrated that in the absence of the templates no dendritic nanomaterials are produced, therefore confirming that the AuAg NWs are necessary in this synthesis. Finally, we demonstrated that the Pt and RhPt dendritic NWs exhibit excellent catalytic performances for the electro-oxidation of methanol and formic acid. In both cases the RhPt dendritic NWs out-performed the Pt dendritic NWs producing higher current densities at lower potentials and demonstrated high resistance to catalyst poisoning in the case of methanol oxidation. We believe that these unique nanomaterials are important for the future fuel cell research and development.

## Conflicts of interest

There are no conflicts to declare.

## Supplementary Material

RA-009-C9RA04801D-s001
