# Supplementary material for: Pt and RhPt dendritic nanowires and their potential application as anodic catalysts for fuel cells
Source: RSC Adv. 2019 Oct 2;9(53):31169–76. doi: 10.1039/c9ra04801d (PMC9072555; doi:10.1039/c9ra04801d)
Supplement: RA-009-C9RA04801D-s001 [file RA-009-C9RA04801D-s001.pdf]

Electronic Supplementary Material (ESI) for RSC Advances.
This journal is © The Royal Society of Chemistry 2019

Supporting information

Pt and RhPt dendritic nanowires and their potential application as anodic catalysts for fuel cells

Daniel K. Kehoe, Sarah A. McCarty, Luis Romeral, Michael G. Lyons, Yurii K. Gun`ko

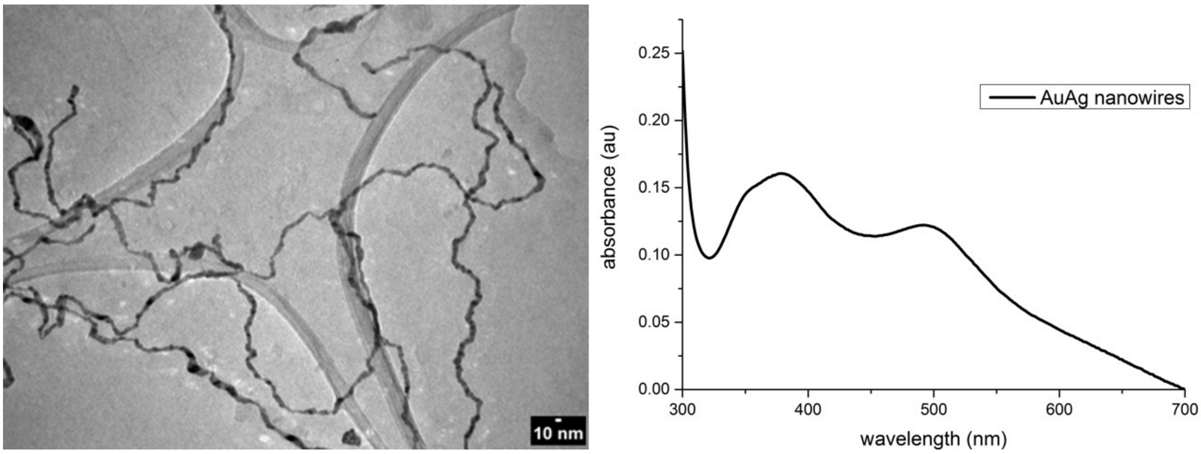

Fig. S1: TEM image (left) and corresponding UV-Vis spectrum of ultrathin AuAg NWs**.**

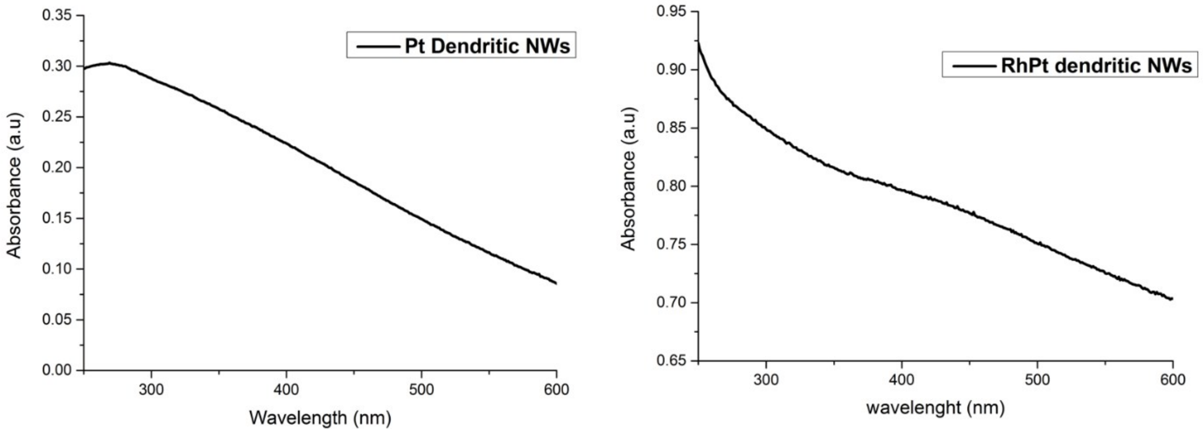

Fig. S2: UV-vis spectra of Pt (left) and RhPt (right) dendritic NWs.

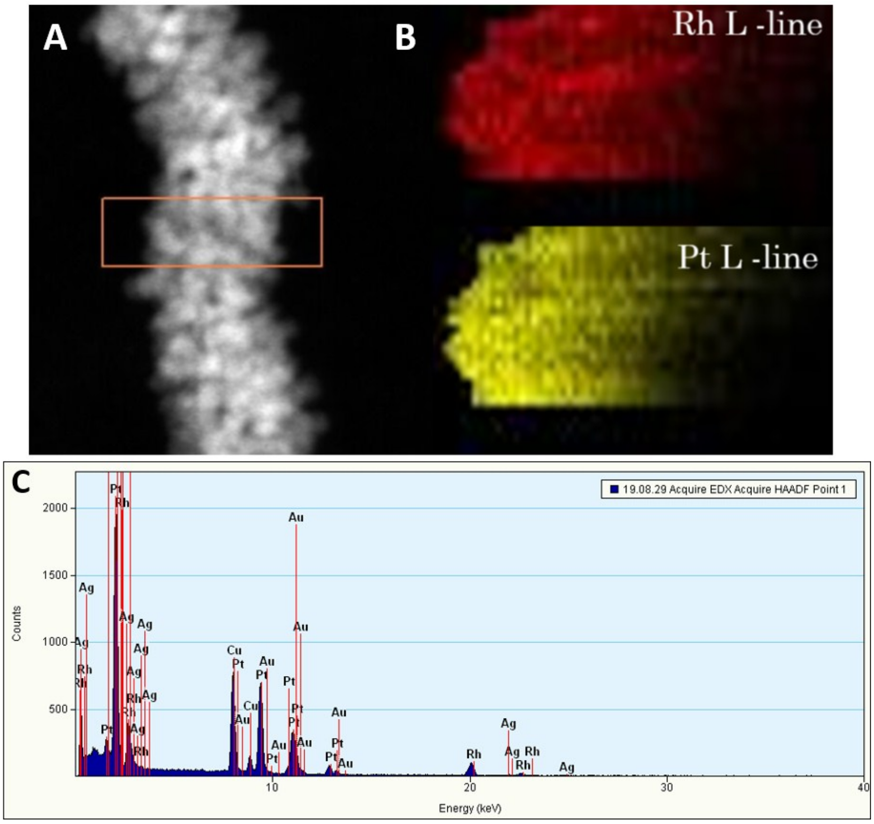

Fig. S3: HAADF STEM image highlighting region of interest (A), EDX maps of Pt and Rh L lines (B) and corresponding EDX spectrum (C) of RhPt dendritic NWs. The red markers indicate positions were the characteristic peaks are expected.

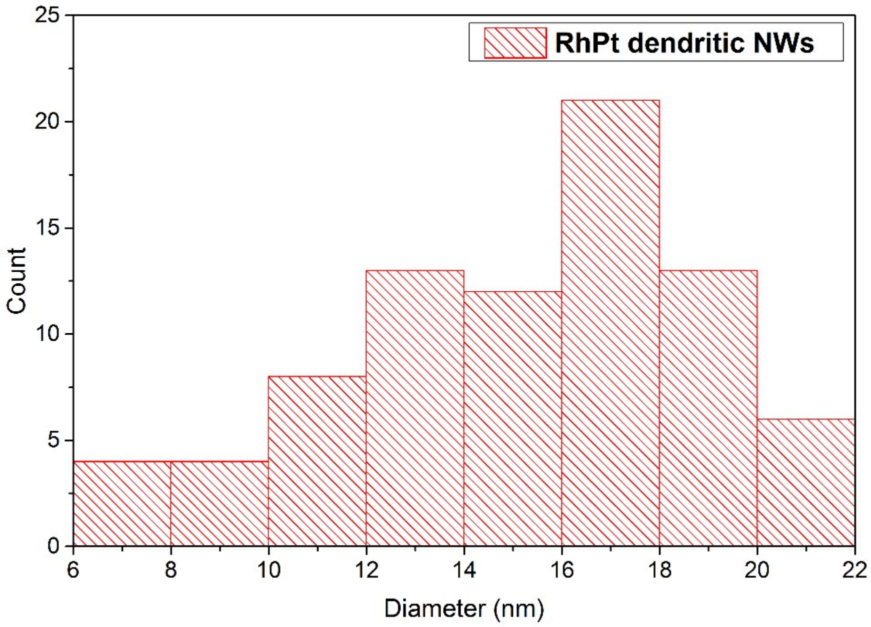

Fig. S4: Size distribution of RhPt dendritic NWs produced after 2.5 mins with average diameter of 15.1 nm.

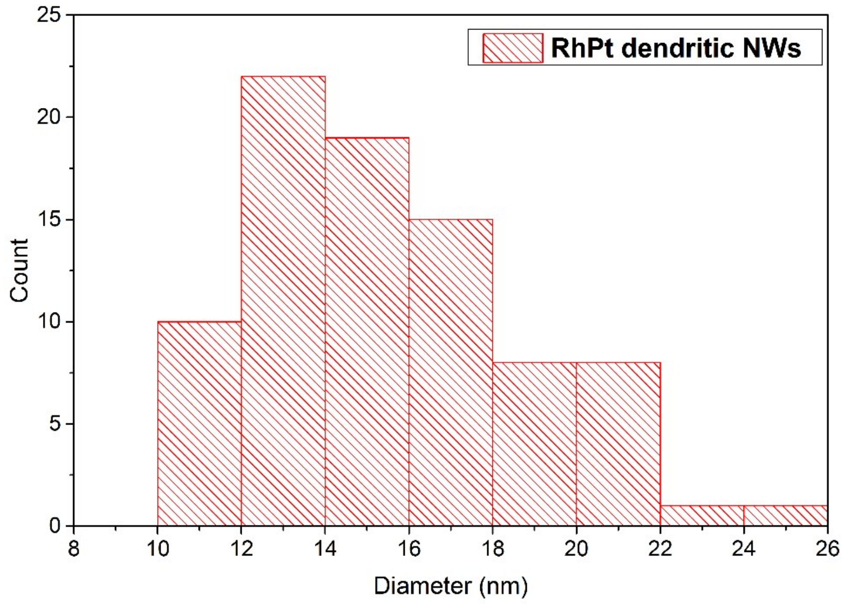

Fig. S5: Size distribution of RhPt dendritic NWs produced after 10 mins with average diameter of 15.5 nm.

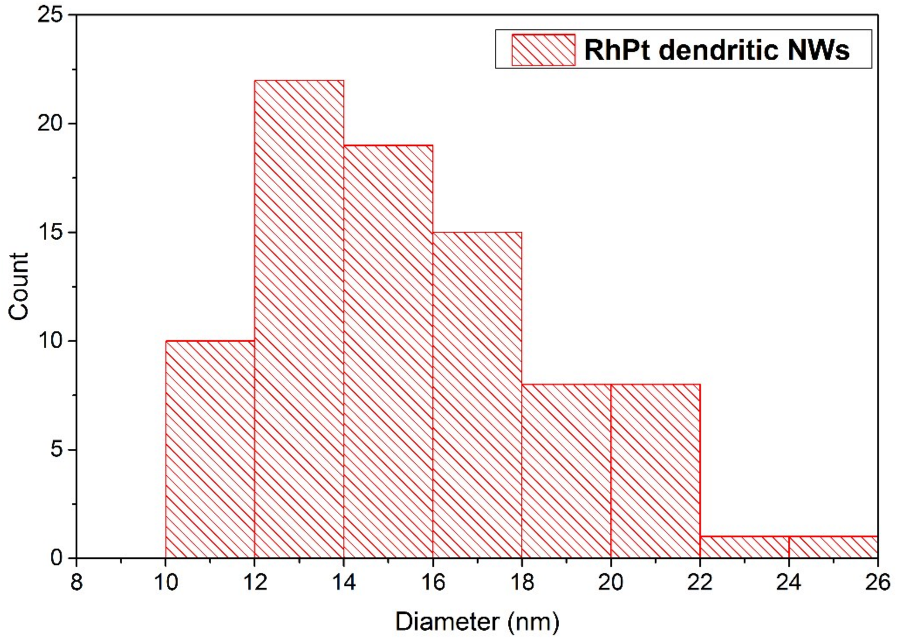

Fig. S6: Size distribution of RhPt dendritic NWs produced after 15 mins with average diameter of 22.4 nm.

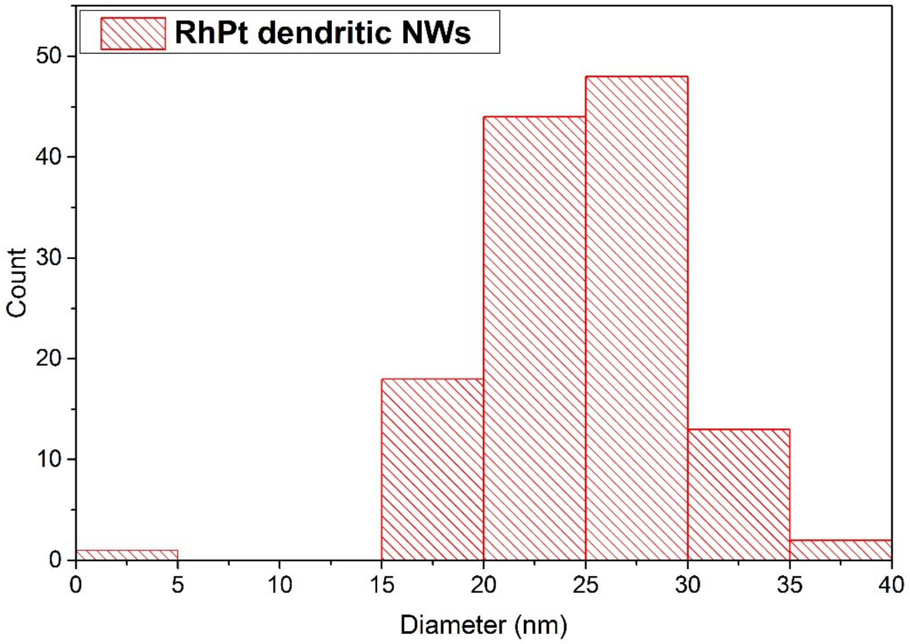

Fig. S7: Size distribution of RhPt dendritic NWs produced after 25 mins with average diameter of 24.7 nm.

.

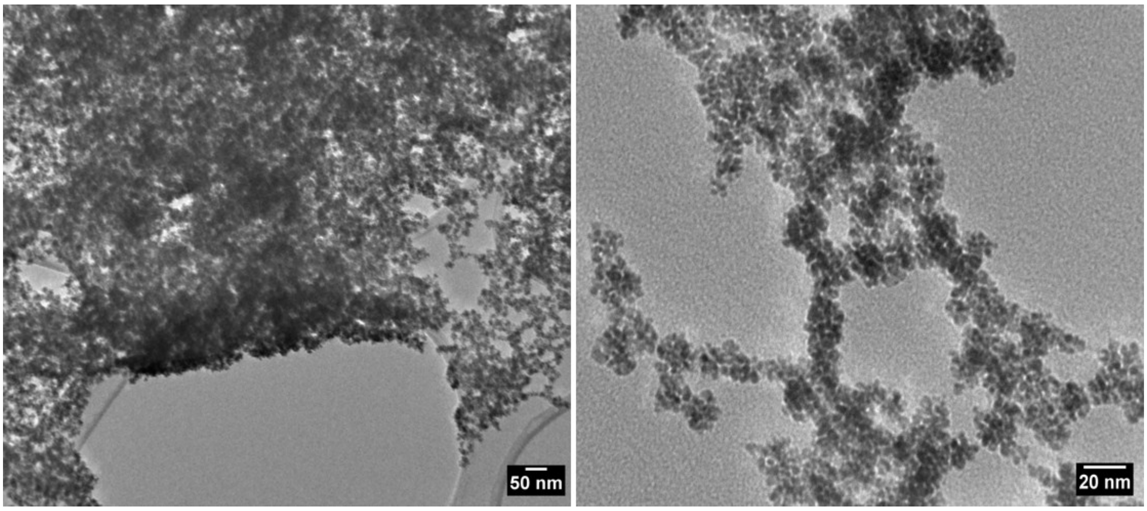

Fig. S8: TEM images of RhPt dendritic NWs synthesis in the absence of PVP.

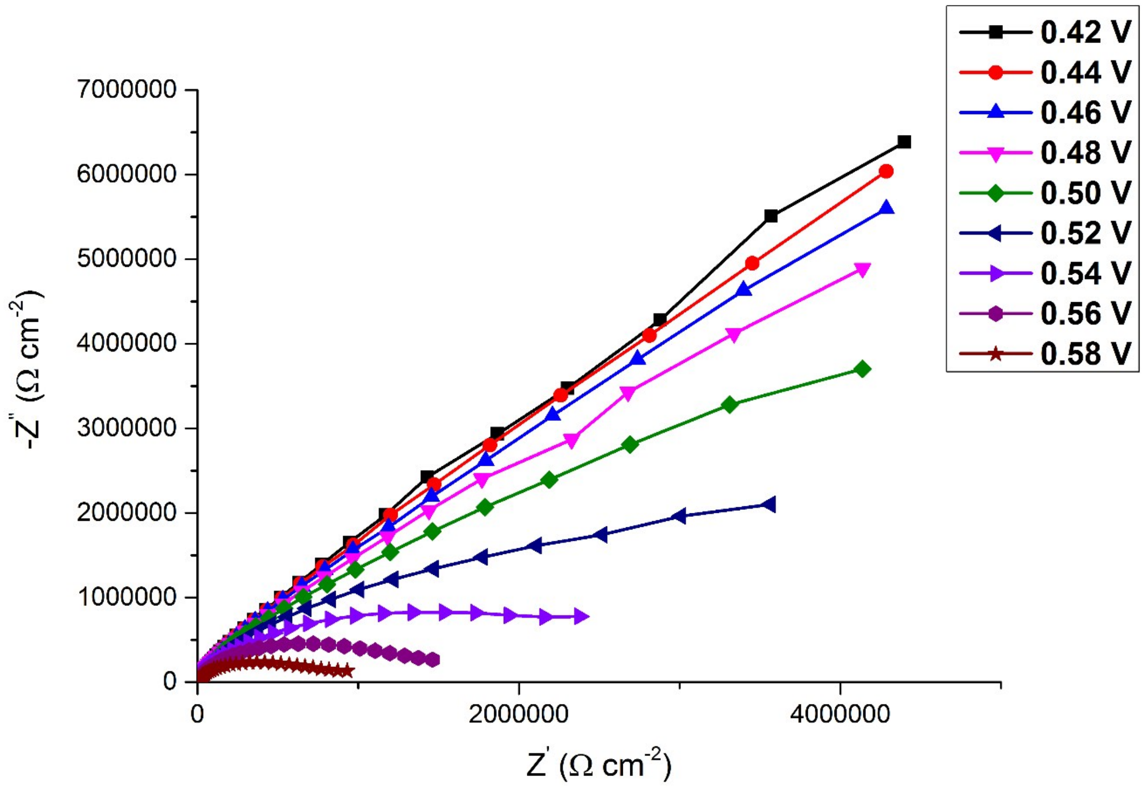

Fig. S9: Nyquist plot of RhPt dendritic NWs in 1M methanol + 0.5 M perchloric acid solution versus the Ag/AgCl reference electrode at 25℃ .

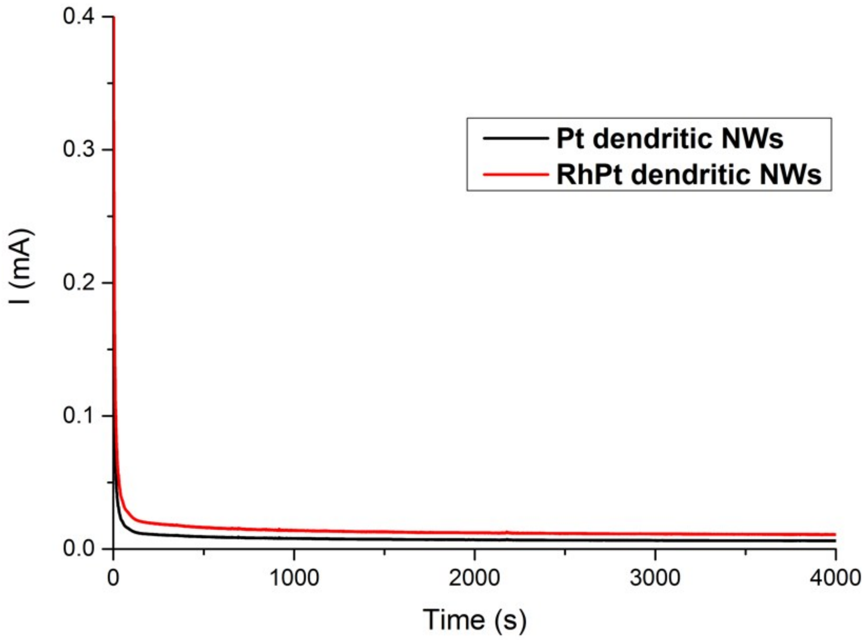

Fig. S10: I-t curve over 4000 s of Pt and RhPt dendritic NWs in N_2-saturated 1 M H_2SO_4 solution containing 0.5 M formic acid.

The electrochemical active surface areas of both catalysts was determined by integrating the area within the hydrogen adsorption region (-0.25 - 0 V) and dividing by the charge density associated with the deposition of a hydrogen monolayer on planar polycrystalline Pt typically 0.21 mC cm^{-2}. ESCA normalised CV analysis for Pt and RhPt dendritic NWs is shown in Figure S6.

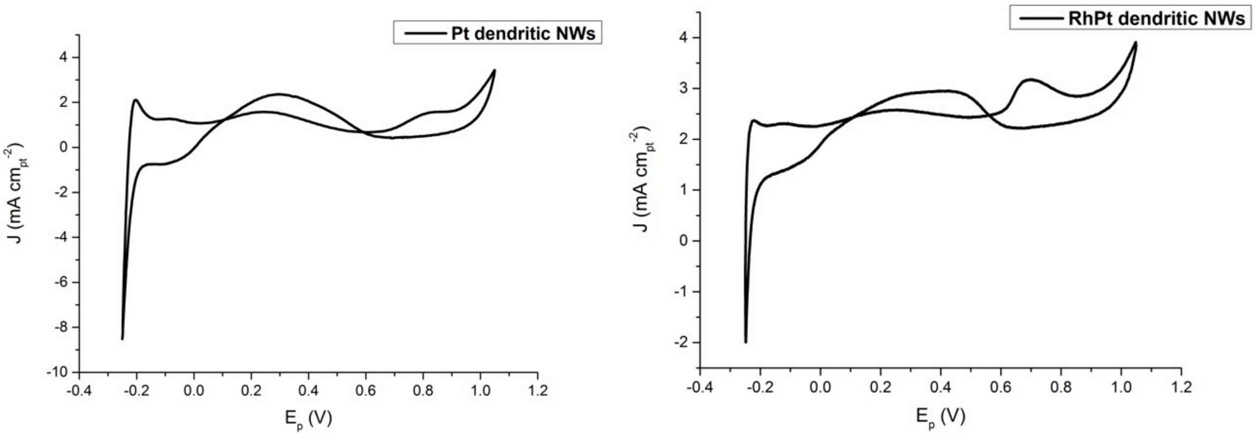

Fig. S8: ESCA normalised CV analysis of formic acid oxidation at 50 mV s^{-1} for Pt (left) and RhPt (right) dendritic NWs versus Ag/AgCl reference electrode in a 1 M H_2SO_4 solution containing 0.5 M Formic acid. The peak current densities in the forward scan was determined to be 1.5 and 3.2 mA cm^{-2} for the Pt and RhPt dendritic NWs respectively.

Table S1: Electro-oxidation of formic acid by various noble metal catalyst in acidic medium.

**Catalyst**	**E_p (V)**	**J (mA cm^{-2})**
Rh nanochains[1]	0.42 (vs Hg/Hg_2SO_4)	0.55
Pt NWs[2]	0.7 (vs sat. Calomel)	1.75
Pt_{71} Au_{29} NWs[2]	0.58 (vs sat. Calomel)	1.2
$Pd_{0.65}$ Ag_1/CNT[3]	*ca.* 0.37 (Hg/HgO)	2.16
PtZn NWs[4] (1.8 nm)	*ca.* 0.63 (vs sat. Calomel)	3.9
Pd_3Ag_1 nanotrees[5]	*ca.* 0.48 (vs RHE)	3.26
PtAg NWs[6]	0.57 (vs RHE)	1.03
Pd NWs[7] (2 nm)	0.64 (vs RHE)	2.4
Pt dendritic NWs (this work)	0.83 (vs Ag/AgCl)	1.5
PtRh dendritic NWs (this work)	0.69 (vs Ag/AgCl)	3.1

CNT = carbon nanotube NP = nanoparticle RHE = relative hydrogen electrode.

References

1. Sathe, B. R.; Balan, B. K.; Pillai, V. K., Enhanced electrocatalytic performance of interconnected Rh nano-chains towards formic acid oxidation. *Energy and Environmental Science* **2011,** *4* (3), 1029-1036.
2. Han, Y.; Ouyang, Y.; Xie, Z.; Chen, J.; Chang, F.; Yu, G., Controlled Growth of Pt–Au Alloy Nanowires and Their Performance for Formic Acid Electrooxidation. *Journal of Materials Science and Technology* **2016,** *32* (7), 639-645.
3. Huang, L.; Yang, J.; Wu, M.; Shi, Z.; Lin, Z.; Kang, X.; Chen, S., PdAg@Pd core-shell nanotubes: Superior catalytic performance towards electrochemical oxidation of formic acid and methanol. *Journal of Power Sources* **2018,** *398* (April), 201-208.
4. Pei, J.; Mao, J.; Liang, X.; Zhuang, Z.; Chen, C.; Peng, Q.; Wang, D.; Li, Y., Ultrathin Pt-Zn Nanowires: High-Performance Catalysts for Electrooxidation of Methanol and Formic Acid. *ACS Sustainable Chemistry and Engineering* **2018,** *6* (1), 77-81.
5. Jiang, X.; Xiong, Y.; Wang, Y.; Wang, J.; Li, N.; Zhou, J.; Fu, G.; Sun, D.; Tang, Y., Treelike two-level PdxAgy nanocrystals tailored for bifunctional fuel cell electrocatalysis. *Journal of Materials Chemistry A* **2019,** *7* (10), 5248-5257.
6. Jiang, X.; Fu, G.; Wu, X.; Liu, Y.; Zhang, M.; Sun, D.; Xu, L.; Tang, Y., Ultrathin AgPt alloy nanowires as a high-performance electrocatalyst for formic acid oxidation. *Nano Research* **2018,** *11* (1), 499-510.
7. Wang, Y.; Choi, S. I.; Zhao, X.; Xie, S.; Peng, H. C.; Chi, M.; Huang, C. Z.; Xia, Y., Polyol synthesis of ultrathin Pd nanowires via attachment-based growth and their enhanced activity towards formic acid oxidation. *Advanced Functional Materials* **2014,** *24* (1), 131-139.
